# Preparation and Properties of *sc*-PLA/PMMA Transparent Nanofiber Air Filter

**DOI:** 10.3390/polym10090996

**Published:** 2018-09-06

**Authors:** Shengnan Lv, Xin Zhao, Lei Shi, Gaokai Zhang, Shubo Wang, Weimin Kang, Xupin Zhuang

**Affiliations:** 1 State Key Laboratory of Separation Membranes and Membrane Processes, Tianjin Polytechnic University, Tianjin 300387, China; lvshnan112358@163.com; 2 School of Textile, Tianjin Polytechnic University, Tianjin 300387, China; shilei@tjpu.edu.cn (L.S.); zgk846045649@outlook.com (G.Z.); shuboever@163.com (S.W.); kangweimin@tjpu.edu.cn (W.K.); 3 School of Science, Tianjin Polytechnic University, Tianjin 300387, China; zhaoxinever@126.com

**Keywords:** air filtration, PM_2.5_, nanofibers, optical properties, mechanical properties, solution blowing

## Abstract

Particulate matter (PM) pollution is a serious concern for the environment and public health. To protect indoor air quality, nanofiber filters have been used to coat window screens due to their high PM removal efficiency, transparency and low air resistance. However, these materials have poor mechanical property. In this study, electrostatic induction-assisted solution blowing was used to fabricate polylactide stereocomplex (*sc*-PLA), which served as reinforcement to enhance the physical cross-linking point to significantly restrict poly(methyl methacrylate) (PMMA) molecular chain motion and improve the mechanical properties of *sc*-PLA/PMMA nanofibers. Moreover, the introduction of *sc*-PLA led to the formation of thick/thin composite nanofiber structure, which is beneficial for the mechanical property. Thus, *sc*-PLA/PMMA air filters of ~83% transparency with 99.5% PM_2.5_ removal and 140% increase in mechanical properties were achieved when 5 wt % *sc*-PLA was added to PMMA. Hence, the addition of *sc*-PLA to transparent filters can effectively improve their performance.

## 1. Introduction

Particulate matter (PM) is a major air pollutant that threatens the environment and public health. PM_2.5_, with aerodynamic diameters smaller than 2.5 *µ*m, has been associated with detrimental health outcomes, such as cancer, respiratory illnesses and cardiovascular diseases [1,2,3,4,5,6]. Source control and air purification are the two main measures used to solve the PM pollution problem, with the latter being considered the most feasible and effective way for outdoor individual protection [7]. Under hazardous air conditions, residents are forced to close windows and doors or turn on air cleaners to block outdoor PM. According to a survey, Beijing’s residential ventilation level is only 0.17 h^−1^, which is far below China’s national standard of 1.0 h^−1^ and is not conducive to good health as a result of insufficient indoor air circulation [8]. To overcome this challenge, a novel strategy should be developed to reduce PM_2.5_ and provide sufficient ventilation.

Fibrous air filters capture PM through physical barriers and surface adhesion through inertial impaction; interception; Brownian motion; or gravitational, chemical and electrical forces [9,10,11]. With the rapid development of fiber fabrication technologies, nanofiber air filters with fiber diameter of dozens or even hundreds of nanometers have high PM filtration efficiency and low air flow resistance [8]. Ding’s research group fabricated polyamide-56 nanofiber/net filters with high filtration efficiency of 99.995% [12]. Wang et al. created electrospun silk nanofiber filters as lightweight air filters with high PM filtration efficiency of 99% [13]. Wang and Pan developed hierarchically structured nanosized/porous poly(lactic acid) composite fibrous filters for air filtration with 99.999% removal efficiency [14,15]. Recently, Cui et al. coated polymer nanofibers onto window screens and developed a transparent nanofiber air filter that can effectively filter out a majority of outdoor PM_2.5_ in the incoming air and maintain efficacious air exchange rate during natural ventilation because of its relatively low air resistance [7]. The polyacrylonitrile (PAN) nanofiber filter with a transmittance of ~77% has a PM_2.5_ removal efficiency of 98.69%. Khalid et al. and Zhang et al. further fabricated polyacrylonitrile and polyimide nanofibers with high productivity and thermal stability [16]. These kinds of transparent air filter screen (TAFS) open up new ways of energy-free protection of indoor air quality though natural passive ventilation.

Optical transmittance is an important characteristic of TAFS because it allows sunlight transmission. Reflections and refractions can occur at the fiber interface, as well as at the crystalline and amorphous interfaces within the fiber, because the medium for light propagation changes, thereby reducing transmittance [17]. Scattering is another important factor affecting transmittance [18]. Poly(methyl methacrylate) (PMMA), a widely applied transparent polymer, can improve the transmittance in TAFS. However, amorphous PMMA chains will irreversibly slide with one another during deformation, inevitably leading to their poor mechanical performance [19]. Generally, nanofillers include multiwalled carbon nanotubes, graphene oxide and SiO_2_ to enhance their mechanical properties [20,21,22,23]. However, these components inevitably lead to the reduction of optical properties due to the aggregation of the fillers [19].

Polylactide (PLA), a biodegradable and biocompatible thermoplastic polymer, possesses good mechanical and optical properties. Interestingly, enantiomeric poly(*l*-lactide) (PLLA) and poly(*d*-lactide) (PDLA) can form the stereocomplex (*sc*)-PLA, which not only possesses high melting temperature (*T*m) but also excellent optical properties and improved mechanical performance [19,24,25,26]. In a previous report, the mechanical property of *sc*-PLA/PMMA membrane by melt blending increased by 50% compared with that of pure PMMA [19] with the help of the crystalline *sc*-PLA acting as a physical cross-link in the PMMA matrix [19,27]. Thus, *sc*-PLA is supposed to be an ideal filler to enhance the mechanical performance of PMMA nanofibers while maintaining its optical transparency. However, whether *sc*-PLA and PMMA nanofibers are miscible between components is a prerequisite. Miscibility in polymer-polymer systems is principally governed by the heat of mixing; pairs of polymers whose analogues exhibit a negative heat of mixing are miscible and generally require some favorable interactions, such as hydrogen bonding, donor–acceptor interaction and charge transfer, resulting in a negative exchange interaction contribution to the free energy of mixing [28,29,30]. The PLLA/PDLA/PMMA blends show partial miscibility because some kind of weak dipolar interaction can take place owing to the chemical structure of the polymers [19,25,31,32,33]. To further improve their miscibility, electrostatic induction-assisted solution blowing (ESB), as we reported previously, was applied [34], with the assumption that the electrostatic field can help form *sc*-PLA and is beneficial to the miscibility of the polymers. Interestingly, optically transparent *sc*-PLA/PMMA nanofiber air filters with significantly enhanced mechanical properties were fabricated in our study and the introduction of *sc*-PLA resulted in thick/thin composite nanofibers, which enhanced the PM removal efficiency.

## 2. Materials and Methods

### 2.1. Materials

PMMA (molecular weight (*M*_w_) = 3.0 × 107 g/mol, refractive index *n* = 1.49) was supplied by Qimei Co., Ltd. (Taiwan, China). PLLA and PDLA with *M*_w_ of 3.0 × 105 g/mol was purchased from NatureWorks Co., Ltd. (Blair, NB, USA). 1,1,3,3,3-Hexafluoro-2-propanol (HFIP) were purchased from Weng Jiang Chemical Reagents Co., Ltd. (Guangdong, China).

### 2.2. ESB of sc-PLA/PMMA Nanofibers

**Figure 1:**
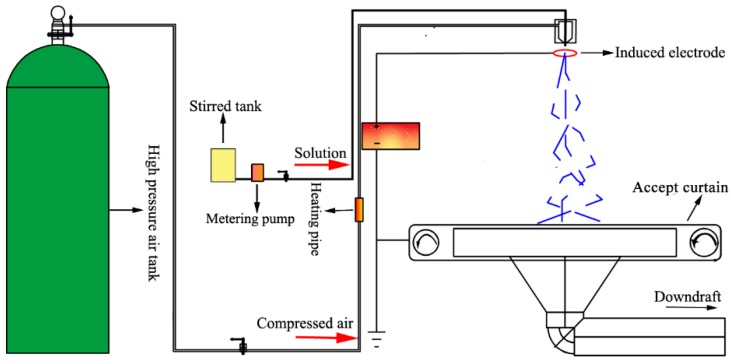
Schematic of the preparation of transparent air filter material by electrostatic induction-assisted solution blowing.

Spinning solutions were prepared by dissolving PMMA and various amounts of PLA (PLLA:PDLA = 1:1 in weight) in HFIP. Then, *sc*-PLA/PMMA nanofiber filters were fabricated by ESB [34] with a self-made equipment (Figure 1). The spinning parameters were as follows: induction voltage of 20 kV, air pressure of 0.1 MPa, solution feed rate of 10 mL/h and collect distance of 40 cm. The ESB nanofibers were collected using a grounded copper mesh with size of 2 × 3 mm. The PLLA/PDLA contents, which account for PMMA in weight, were used to mark the final *sc*-PLA/PMMA nanofibers. X% means that *sc*-PLA is the X% of the quality of PMMA.

### 2.3. Characterization

#### 2.3.1. Scanning Electron Microscopy (SEM)

Nanofiber morphologies were observed using an SEM (Hitachi S-4800, Hitachi Limited Co., Ltd., Tokyo, Japan). Fiber diameter was determined from SEM micrographs using Image-Pro Plus (Ipwin32, Soft Imaging System, Pro 6.0, Media Cybernetics Co., Ltd., Washington, USA).

#### 2.3.2. Transmission Election Microscopy (TEM)

The morphology of the nanofiber mats was investigated using a TEM (Hitachi H7650, Hitachi Limited Co., Ltd., Tokyo, Japan) with acceleration voltage of 100 kV.

#### 2.3.3. Differential Scanning Calorimetry (DSC)

Thermal analysis of nanofiber mats was carried out using a DSC (DSC204F1, NETZSCH Scientific Instruments Trading Ltd., Selb, Germany). Samples with mass of 5–10 mg were heated under nitrogen environment at 60–240 °C, which increased at 10 °C/min.

#### 2.3.4. Rheological Measurements

Rheological measurements were performed using a Bohlin CVO rheometer (Malvern Instruments Co., Ltd., Malvern, UK). Dynamic oscillatory shear measurements were conducted by using a set of 40 mm-diameter parallel plates at 25 °C in the frequency range of 0.1–10 rad/s.

#### 2.3.5. Mechanical Performance Measurement

Mechanical performance was determined with an electronic monofiber strength tester (LLY-06, Laizhou Electronic Instrument Co., Ltd., ShenZhen, China). Nanofiber mats had a size of 20 mm × 50 mm and the tests were performed at a strain rate of 10 mm/min. The pinch distance was 10 mm.

#### 2.3.6. Optical Properties of Nanofiber Filter

The optical properties of the nanofiber filter were measured with a Lambda 750 spectrophotometer (PerkinElmer, Boston, MA, USA). The spectrum ranged from 700 to 400 nm with a scanning step of 2 nm. The following linear formula based on simple optical transmission was used to calculate the transmittance of nanofiber filters (Equation (1)):

*I*_f_ = *I*_T_/*I*_s_ (1)

where *I*_T_ is the transmittance (%) through TFAS, *I*_f_ is the transmittance through the nanofiber filter and *I*_s_ is the transmittance through copper mesh support.

#### 2.3.7. Measurement of Removal Efficiency

The removal efficiency for aerogel particles with different mass median diameters and the air permeability of the nanofiber filters were analyzed with a filter material test bed (AFC 131, TOPAS Co., Ltd., Dresden, Germany). The *n*-butanol (Weng Jiang Chemical Reagents Co., Ltd. (Guangdong, China)) uptake tests were performed to investigate the porosities of those membranes according to the Equation (2):

Porosity (%) = (*W*_w_ − *W*_d_)/(*ρ*_b_ × *V*) (2)

where *W*_w_ is the weight of the wet membrane and *W*_d_ is the weight of the dry membrane; *ρ*_b_ and *V* are the density of n-butanol and the geometric volume of the membrane, respectively.

## 3. Results and Discussion

### 3.1. Formation of sc-PLA in PMMA Nanofibers

**Figure 2:**
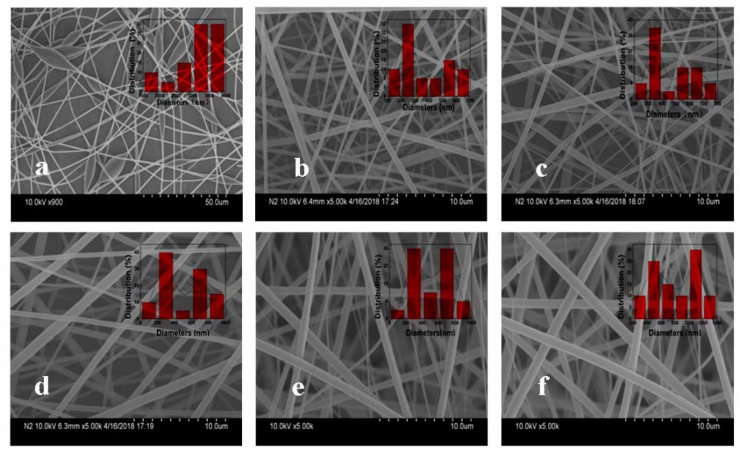
SEM micrographs and diameter distributions of the sc-PLA/PMMA nanofibers with *sc*-PLA contents of (**a**) 0 wt %, (**b**) 5 wt %, (**c**) 10 wt %, (**d**) 15 wt %, (**e**) 20 wt % and (**f**) 25 wt %.

Solution blowing is a quasi-commercial method to produce nanofibers with high efficiency and electrostatic induction is beneficial in obtaining a homogeneous fiber distribution, as found in our previous work [34]. The electrostatic field is an effective means to separate fiber bundles formed by airflow during solution blow spinning. ESB is an important method for mass production of nanofiber membranes [35]. The high porosity and small pore size of nanofiber membranes make them good candidates for filters [36]. In addition, the morphology of the fiber also determines the filtration effect of the fiber filter. Figure 2 shows the morphology of PMMA and *sc*-PLA/PMMA nanofibers. The figure shows that PMMA nanofibers from 8 wt % solution do not have good morphology and some beads are observed (Figure 2a). When PLLA/PDLA was added to the PMMA, the fibers had good morphology and smooth surface (Figure 2b–f). Interestingly, the addition of PLLA/PDLA led to the formation of a composite structure with thick and thin fibers. The nanofibers showed bipolar distribution in diameter. For example, with 5 wt % PLLA/PDLA, the number of thin fibers was higher than that of thick fibers. The diameter of thin fibers was 250 nm, while that of thick fibers was 550 nm (Figure 2b). As the amount of PLLA/PDLA increased, the diameters of the thin and thick fibers increased. The average diameter of the thin fiber increased from 250 to 550 nm and that of thick fibers increased from 550 to 1300 nm. Meanwhile, the difference between the diameters of the thick and thin fibers significantly increased and the amount of thin fibers gradually decreased. When 25 wt % PLLA/PDLA was added, a few thin fibers were observed; the diameter of the thin fibers was 400–600 nm and that of the thick fibers was 1200–1400 nm. The results showed that nanofibers can be obtained successfully by ESB from the blend solutions and that the addition of PLLA/PDLA leads to thick/thin composite nanofibers, which may influence the mechanical property, removal efficiency and optical transmittance.

**Figure 3:**
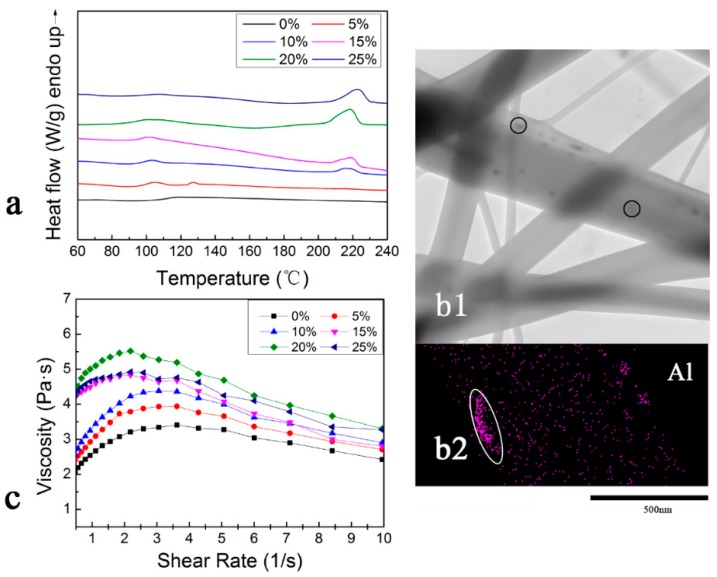
Analysis of the fabrication of *sc*-PLA in PMMA. (**a**) DSC melting curves for the different samples with various *sc*-PLA contents. (**b1**) TEM images for *sc*-PLA/PMMA nanofibers with 20 wt % *sc*-PLA contents. (**b2**) EDS mapping of aluminum. (**c**) Change of viscosity with shear rate for various *sc*-PLA contents.

Recently, *sc*-PLA nanofibers have been successfully fabricated through electrospinning and high-voltage electrostatic field is proven to enhance the formation and growth of *sc*-crystallites [37]. Therefore, *sc*-PLA is expected generation in the PMMA nanofibers with the aid of induced electric field in the ESB process. DSC was used to determine the thermal properties of the nanofibers, as shown in Figure 3a. In the present study, the following thermal properties were observed: all nanofibers exhibited glass transition of PMMA at around 105 °C. The blend nanofibers with over 10 wt % *sc*-PLA showed obvious endothermic peaks at 210–250 °C, which is consistent with the melting peak of *sc*-PLA reported in the literature [38]. No melting peak of PLLA/PDLA homo-crystallization at around of 170 °C was observed for all the blend nanofibers. *T*m of *sc*-PLA was reported to be 30–60 °C higher than that of individual PLLA and PDLA, which is attributed to the close packing of polymer chains, strong intermolecular interactions and consequently increased crystallinity [38,39,40,41] As shown in Figure 3a, the blends containing more than 5 wt % of *sc*-PLA show only one melting point at about 215 °C. This indicates electrostatic field applied in ESB process favor the formation of *sc*-PLA in PMMA nanofibers and is beneficial to improve the miscibility of *sc-*PLA and PMMA under certain conditions of *sc*-PLA content [42].

TEM analysis is performed to investigate the microstructure of the *sc*-PLA/PMMA nanofibers. As shown in Figure 3b, the thin nanofibers exhibit a homogeneous phase, whereas some blocks (encircled in Figure 3(b1)) are observed in the thick fibers. EDS mapping is used to study the composition of blocks in thick fibers. Aluminum is found to be aggregated in the blocks of the fibers, encircled in Figure 3(b2). Aluminum alkyl is commonly used in ring-opening polymerization of lactide as a catalyst; hence, the aggregated blocks are inferred as *sc*-PLA crystalline. That is to say, *sc*-PLA only exists in thick fibers or increases the diameter of the fiber.

The rheological behavior of the spinning solutions of PMMA and the blend solutions are further investigated, as shown in Figure 3c. The rheological properties can reflect the viscoelastic behaviors of the polymer solution and are highly sensitive to the interactions and structure within the polymer solution [43,44,45]. As Cui reported, shear force during solution stirring process helps PLLA and PDLA molecular chains to contact effectively and facilitate the formation of *sc*-PLA [46]. PMMA molecular chain movements are constrained by PLLA/PDLA, *sc*-PLA, or their aggregations due to the entanglements of PMMA molecular chains with PLLA/PDLA or *sc*-PLA, thereby gradually increasing internal flow friction, deformation resistance and solution viscosity [47]. As the amount of added *sc*-PLA increases, the viscosity of the solution gradually increases (Figure 3c). Moreover, with the addition of PLLA/PDLA, the curves become tortuous and not as smooth as the line of PMMA solution, which indicates that the blend solutions are heterogeneous and have poor miscibility at ambient temperature [42,48]. The characteristic of heterogeneous solution is regarded as the reason for the formation of different jets in spinning and thick/thin composite nanofibers.

### 3.2. Performance of sc-PLA/PMMA Nanofiber Filters

#### 3.2.1. Mechanical Properties

**Figure 4:**
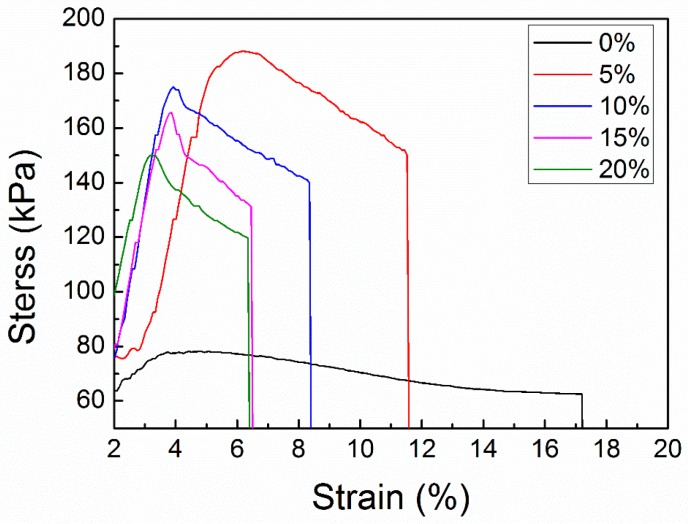
Stress–strain curves of *sc*-PLA/PMMA nanofiber filter with different contents of *sc*-PLA.

Restricting the chain movement of PMMA polymers can improve their tensile strength Molecular mobility has been claimed to be the factor governing the mechanical property and cross-links are responsible for effecting molecular mobility [49]. Herein, *sc*-PLA is introduced to enhance the mechanical property of PMMA nanofibers, considering that *sc*-PLA can serve as a reinforcement to enhance the physical cross-linking points to markedly confine the polymer chain motion and facilitate good interaction between the PMMA matrix and the *sc*-PLA [19,50]. Figure 4 shows the tensile stress–strain curves of PMMA nanofibers and *sc*-PLA/PMMA nanofibers with different composition ratios of *sc*-PLA and all curves present the same profile. The tensile strength of PMMA nanofibers was only 75 kPa, while that of *sc*-PLA/PMMA increased significantly. It is worth noting that all *sc*-PLA/PMMA nanofibers exhibited a breaking strength over 150 kPa with the introduction of *sc*-PLA. This indicates that the formation of *sc*-PLA prominently improved the mechanical property effectively. The results are in accordance with those in a previous works [19] and the reason is ascribed to the presence of a high density of intercrystalline connections through a mobile amorphous phase, which simultaneously enhanced the strength and elongation at break simultaneously [26]. It is well known that the mechanical properties of nanofiber filters depend on the molecular arrangement [42]. The blended nanofiber filter with 5 wt % *sc*-PLA exhibited the highest breaking strength and elevated s*c*-PLA loading demonstrated an opposite change tendency. This may be attributed to the fact that the mechanical property does not depend too much on the cross-links between *sc*-PLA and PMMA and high *sc*-PLA crystallization in nanofibers may weaken the connections in the PMMA amorphous phase.

#### 3.2.2. Optical Transmittance

**Figure 5:**
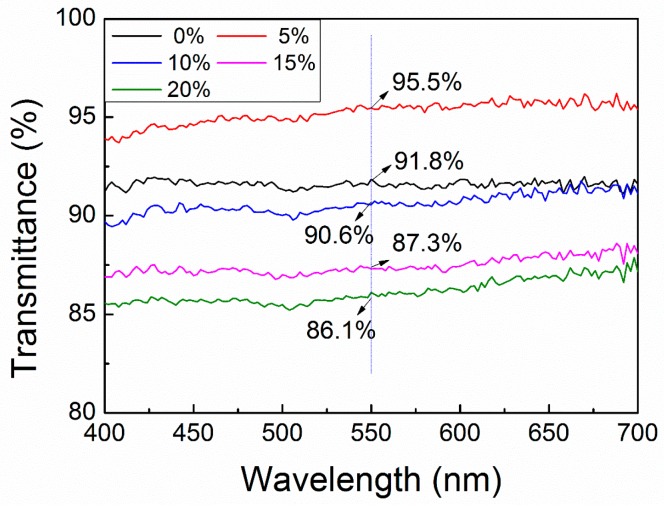
Transmittance of *sc*-PLA/PMMA nanofiber filter with different *sc*-PLA contents.

The transparency of nanofiber filters, which is significant in practical applications, was tested with different composition ratios and same weight (approximately 2 g/m^2^). The results are shown in Figure 5. Although PMMA nanofibers exhibited poor configuration, they had high transparency within the range of visible wavelength (400–700 nm), which could reach up to 91.8% at 550 nm. Conversely, the nanofibers containing 5 wt % *sc*-PLA demonstrated higher transparency throughout the visible wavelength with 95.5% transmittance at 550 nm. However, their optical transparency was slightly reduced with the increase in *sc*-PLA; the transmittance at 550 nm was reduced to 90.6%, 87.3% and 86.1% for the nanofibers containing 10, 15 and 20 wt % of *sc*-PLA, respectively. The results indicate that optical transparency can be improved with a suitable content of *sc*-PLA, while excess *sc*-PLA produces a negative effect, which might be attributed to the increase in thick fibers with increasing *sc*-PLA.

#### 3.2.3. PM Removal Efficiency

**Figure 6:**
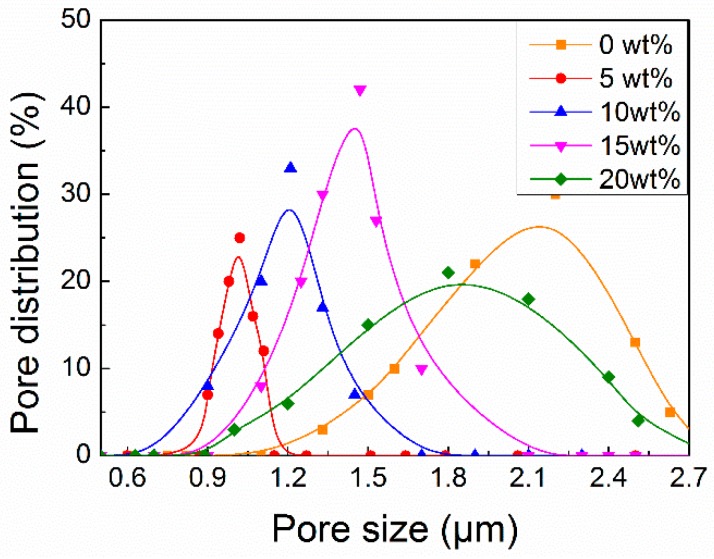
Pore distribution of *sc*-PLA/PMMA nanofiber filter with different *sc*-PLA contents.

Aside from the ability of *sc*-PLA/PMMA filters to transmit sunlight, their ability to capture PM particles, another significant parameter, was evaluated. The *sc*-PLA/PMMA filters with different *sc*-PLA contents, having the same transparency of approximately 83% and thickness of approximately 0.02 µm, were selected and their pore structures are shown in Figure 6. All filters demonstrated a concentrated pore size distribution between 0.6 and 2.7 µm and a single distribution peak. As mentioned above, the addition of *sc*-PLA increased the number of thick fibers. Therefore, the average pore size gradually increased as the amount of addition increased; however, the pure PMMA filter had a large average pore diameter due to poor spinning morphology (Figure 2). PM_2.5_ removal efficiency of the *sc*-PLA/PMMA filters was tested with a AFC-131 filtering equipment at a flow rate of 0.2 m/s. As shown in Table 1, the PM_2.5_ removal efficiencies of nanofiber filters with *sc*-PLA contents of 5, 10, 15 and 20 wt % are 99.52%, 99.95%, 99.99% and 96.35%, respectively, which are higher than those of PMMA nanofiber filter (95.40%) and standard high-efficiency filters (95%). As reported in a previous work, diffusion filtration, especially in low velocity flow field, helps increase the adequate interaction between fibers and particles and improve the removal efficiency of particles [51]. *sc*-PLA/PMMA nanofiber filters generated varying diameters and the thin fibers showed obvious diffusion filtration effect. The high porosity (>70%) induced the filter with powerful air permeability. The pressure drops of nanofiber filters with *sc*-PLA contents of 5, 10, 15 and 20 wt % were 46, 65, 67 and 35 Pa, respectively. The low-pressure drop is attributed to the novel structure of thick/thin composite fibers, in which the thick fibers are considered as scaffold between the fibers to provide obvious air permeability effect. Those results exactly reflect the fact that, the most penetrating particle generated by thin fibers is smaller than thick fiber as a filter and lead to higher filtration efficiency that has reported in previous works. Nevertheless, the pressure drop of the filter composed of thin fibers becomes larger as the square of the fiber radius decreases, so the thick fiber can be used to reduce pressure drop. The thick/thin structure in this study is more advantageous for balancing the filtration efficiency and pressure drop [51,52]. The quality factor (*Q*_F_), which is a compromise parameter between removal efficiency and pressure drop, was further used to evaluate the filtration performance of the nanofiber filters with different structure. The higher the *Q*_F_, the better the filter. As shown in Table 1, *sc*-PLA/PMMA filters with different *sc*-PLAs exhibit different *Q*_F_ values and the one with 15 wt % *sc*-PLA obtained the highest *Q*_F_ of 0.1374, which indicates a high-level performance [16].

## 4. Conclusions

In this study, ESB was used to fabricate *sc*-PLA/PMMA nanofibers for transparent air filters. The introduction of *sc*-PLA improved the morphology of the nanofibers and resulted in thick/thin composite nanofiber structure. With 5 wt % *sc*-PLA loading content, the breaking strength of blend nanofibers increased by 140% and a high PM_2.5_ removal efficiency of 99.5% and high optical transmittance of 83% were obtained. Considering the high efficiency of the ESB process, the *sc*-PLA/PMMA nanofiber filters can be applied in TAFS.

## Figures and Tables

**Table 1:** Performance summary of different air filters.

Sample	P (%)	E (%)	ΔP (Pa)	*Q*_F_ (Pa^−1^)
0 wt %	80	95.40	32	0.0962
5 wt %	81	99.52	46	0.1161
10 wt %	75	99.95	65	0.1169
15 wt %	72	99.99	67	0.1374
20 wt %	73	96.35	35	0.0966

Note. P, porosity; E, PM_2.5_ removal efficiency; ΔP, pressure drop; *Q*_F_, quality factor. *Q*_F_ = −ln(1 − E)/ ΔP.
